# Letter from Dr. Carson

**Published:** 1867-08

**Authors:** Wm. Carson

**Affiliations:** Cincinnati, Ohio


We publish the following letter, accompanied by a blank, for the report of any cases of Hepatic Abscess that may have presented itself. Feeling a desire to aid and encourage enterprising investigators, we do so, hoping that any of our readers who may have had charge of such cases will report them to us, so that we can arrange them on the blanks furnished.

The name, age, color, sex and residence of the patient is requested. Also, whether the Abscess was encysted; their position and number; how the matter was discharged; the symptoms attending, and result:—

CINCINNATI, OHIO.

Dear Dr.———

Will you fill up the enclosed blank and return it to me at your earliest convenience. Your report will be suitably acknowledged in my paper on the subject, which I desire to make up as quickly as I can.

Respectfully,
DR. WM. CARSON,
Cincinnati, Ohio.

